# Cancer–Alzheimer inverse comorbidity from epidemiology to protein self-assembly

**DOI:** 10.3389/fendo.2026.1992522

**Published:** 2026-10-19

**Authors:** Yanxian Zhang, Lijin Wang, Jialun Wei, Nazmul Shuzan, Jie Zheng

**Affiliations:** 1 Division of Endocrinology and Diabetes, Department of Pediatrics, School of Medicine, Stanford University, Stanford, CA, United States; 2 Department of Biomedical Engineering and Chemical Engineering, The University of Texas at San Antonio, San Antonio, TX, United States

**Keywords:** Alzheimer’s disease, amyloid self-assembly, cancer, endocrine signaling, proteostasis

## Abstract

Cancer and Alzheimer’s disease (AD) are common age-associated disorders characterized by contrasting cellular outcomes, yet epidemiological studies repeatedly report an apparent, modest bidirectional inverse association between cancer and dementia, including AD. This Mini Review examines the extent to which this pattern reflects biological antagonism versus competing mortality, diagnostic bias, survivor selection, and treatment exposure. Breast and prostate cancers are considered as endocrine models because estrogen- and androgen-receptor signaling supports tumor growth while also regulating neuronal metabolism, mitochondrial function, vascular homeostasis, and inflammation. Population studies show heterogeneous neurological associations for breast cancer hormone-modulating therapy and prostate cancer androgen-deprivation therapy, emphasizing the need to distinguish tumor biology from systemic endocrine treatment. Mechanistically, cancer and AD engage overlapping metabolic pathways, including insulin/IGF–PI3K–AKT, mTOR, and AMPK; cell-fate programs involving cell-cycle control and senescence; and proteostatic systems involving molecular chaperones and autophagy–lysosomal function. These networks produce divergent outcomes: proliferative and proteotoxic adaptation in tumors versus energetic failure, impaired proteostasis, and neuronal degeneration in AD. Protein self-assembly provides an additional, context-dependent connection: amyloidogenic proteins can modify cancer-cell fitness, while recent preclinical evidence identifies a tumor-derived cystatin C–TREM2 pathway that enhances cerebral Aβ clearance. Whether these mechanisms contribute to human cancer–AD inverse comorbidity remains unproven. The observed cancer–AD inverse comorbidity therefore likely reflects interacting epidemiological, endocrine, metabolic, proteostatic, and protein-assembly mechanisms rather than a single causal pathway. Treatment-aware longitudinal studies incorporating AD biomarkers and physiologically relevant models are needed to distinguish genuine biological antagonism from observational bias.

## 1 Introduction

Cancer and Alzheimer’s disease (AD) are prevalent age-associated disorders but represent markedly different cellular trajectories. Cancer cells maintain proliferation through metabolic adaptation, resistance to apoptosis, and enhanced tolerance of genomic and proteotoxic stress. AD, by contrast, is characterized by synaptic dysfunction, impaired energy metabolism, failure of protein quality control, and progressive loss of post-mitotic neurons. Epidemiological studies have repeatedly reported an apparent bidirectional inverse association: individuals with cancer have a modestly lower subsequent incidence of all-cause dementia and, in some studies, AD, whereas people with dementia are less frequently diagnosed with cancer (1, 2). This pattern is neither universal nor necessarily causal, however, because its magnitude varies across cancer types, dementia subtypes, populations, and analytical designs and is influenced by competing mortality, diagnostic surveillance, survivor selection, and the long preclinical phases of both diseases. Hormone-dependent cancers provide particularly informative models for separating intrinsic tumor biology from treatment-related effects. Estrogen- and androgen-receptor signaling promotes the growth of breast and prostate tumors, respectively, while also regulating neuronal metabolism, mitochondrial function, synaptic activity, vascular homeostasis, and inflammation. Endocrine therapies impose sustained systemic hormone perturbations that may affect cognitive and neurodegenerative outcomes independently of the tumor itself. The heterogeneous associations reported for breast cancer hormone-modulating therapy and prostate cancer androgen-deprivation therapy therefore demonstrate why cancer diagnosis, disease severity, and treatment exposure must be evaluated separately (3, 4).

Recent reviews have primarily synthesized epidemiological and Mendelian-randomization evidence or quantified associations between cancer treatments and dementia risk (2, 5). Here, we instead propose a treatment-aware, multilevel framework that separates three distinct questions: the population-level inverse association, the systemic consequences of cancer and its treatment, and the molecular mechanisms that may connect cancer and AD. Our central conclusion is that cancer–AD inverse comorbidity should not be viewed as a single antagonistic biological relationship. Rather, its direction and interpretation depend on the level of analysis: competing mortality and ascertainment influence population estimates; tumor biology and endocrine therapy can produce different systemic effects; and shared metabolic, proteostatic, and protein-assembly pathways generate strongly context-dependent outcomes. Two observations constrain interpretation further. First, cancer associations are not uniform across neurodegenerative disorders or proteinopathies, arguing against a generic cancer–neurodegeneration antagonism. Second, protein self-assembly is not uniformly protective or deleterious: amyloidogenic proteins can modify cancer-cell fitness in experimental models, whereas tumor-derived cystatin C has been shown to enhance TREM2-dependent Aβ clearance in AD models. The latter currently provides the most direct experimentally demonstrated tumor-to-brain mechanism linking cancer with AD pathology. The strength of evidence nonetheless differs across these connections: the inverse associations rest primarily on human epidemiological and neuropathological evidence, whereas most molecular links derive from experimental models, and whether any materially contributes to population-level inverse comorbidity remains unproven. Thus, the key question is not whether one mechanism explains the cancer–AD paradox, but which mechanisms operate in particular cancer, treatment, tissue, and disease contexts. Accordingly, this Mini Review proceeds across four levels of analysis: Section 2 evaluates the population-level epidemiological evidence and its interpretive limitations; Section 3 separates intrinsic tumor biology from treatment-associated endocrine effects; Section 4 examines shared metabolic, cell-fate, and proteostatic pathways; and Section 5 considers protein self-assembly and emerging cancer-to-brain mechanisms.

## 2 Epidemiological evidence and interpretive limits

Population-based evidence generally supports a modest inverse association between cancer and dementia, although effect estimates differ across all-cause dementia, AD, and vascular dementia. In a cohort of 3,021,508 adults aged ≥60 years, a previous cancer diagnosis was associated with a 25% lower subsequent dementia risk (HR 0.75, 95% CI 0.74–0.76) after adjustment for demographic, lifestyle, and cardiometabolic factors. The association persisted after accounting for competing mortality (SHR 0.56, 95% CI 0.55–0.56), while Mendelian-randomization analysis yielded a much smaller inverse association between genetically predicted cancer susceptibility and AD, consistent with—but not proving—a biological contribution beyond selective survival (1). A bidirectional meta-analysis similarly found that cancer survivors had 8–14% lower risks of all-cause dementia, AD (RR 0.88, 95% CI 0.78–0.99), and vascular dementia (RR 0.86, 95% CI 0.75–0.99), whereas individuals with dementia had an approximately 25% lower subsequent cancer risk. These associations varied substantially by cancer site, dementia subtype, treatment exposure, and study quality (5). Nevertheless, the observed association is highly sensitive to disease ascertainment and analytical design. In the UK Biobank, neither prevalent nor incident cancer was significantly associated with AD. All-cause dementia diagnoses increased sharply during the first year after an incident cancer diagnosis but subsequently attenuated to the null, suggesting that intensified healthcare contact, treatment-related cognitive symptoms, or diagnostic investigation can create short-term detection bias (6). In the Rotterdam Study, estimated dementia risk ratios ranged from 0.71 to 1.10 depending on how cancer exposure, confounding, selective survival, and death were handled (7). Competing mortality, reduced cancer screening after dementia, delayed dementia diagnosis after cancer, and the long preclinical phases of both diseases therefore complicate causal interpretation.

Evidence beyond diagnostic records nevertheless suggests that the inverse pattern may contain a biological component. In a community-based autopsy cohort, a history of cancer was associated with lower odds of advanced Braak neurofibrillary tangle stages, neuritic and diffuse plaques, and cerebral amyloid angiopathy, but not with TDP-43, α-synuclein, or cerebrovascular pathology (8). Recent integration of local genetic correlations with transcriptome-wide association analysis identified negatively correlated genomic regions and genes with opposing associations across AD and multiple cancers, converging on cell-cycle regulation, apoptosis, DNA-damage responses, and metabolism (9). Overall, the inverse pattern is modest, cancer-specific, and sensitive to survival, ascertainment, treatment exposure, and the definition of AD outcomes. Inverse cancer associations have also been reported in other neurodegenerative disorders, but they are not uniform. For example, a Korean cohort found a lower overall cancer incidence in patients with Parkinson’s disease (PD), whereas a meta-analysis identified an increased association between PD and melanoma (10, 11). These contrasting patterns argue against a universal cancer–neurodegeneration antagonism. Neuropathological evidence is likewise heterogeneous: although the community autopsy study discussed above found lower AD-type pathology but no association with TDP-43 or α-synuclein (8), a recent NACC analysis reported lower TDP-43 pathology and weaker evidence for lower Lewy body pathology among individuals with cancer (12). Thus, the specificity of the inverse association across neurodegenerative disorders and proteinopathies remains unresolved.

## 3 Hormone-dependent cancers and endocrine therapy

Breast and prostate cancers are emphasized here not as representatives of all malignancies, but as tractable hormone-dependent models in which estrogen- and androgen-receptor signaling is integral to tumor biology and endocrine therapies impose sustained systemic hormonal perturbations. They therefore allow tumor-associated and treatment-associated effects to be considered separately. Broader metabolic and proteostatic mechanisms discussed below may extend across multiple cancer types, whereas sex-steroid-specific mechanisms and endocrine-treatment effects should not be generalized to all malignancies.

### 3.1 Breast cancer: estrogen signaling and endocrine therapy

Breast cancer illustrates how estrogen-dependent tumor biology and estrogen-directed treatment can produce distinct neurological consequences. Estrogen receptor signaling drives the growth and survival of hormone receptor–positive breast tumors, whereas estrogen supports glucose metabolism, mitochondrial function, synaptic plasticity, and inflammatory regulation in the brain. Tumor biology, estrogen deprivation, and treatment-related cognitive symptoms should therefore be distinguished. Population-based studies provide inconsistent evidence that breast cancer survivorship is associated with a lower risk of AD. In a Korean nationwide cohort of 70,701 breast cancer survivors and 180,265 cancer-free controls, survivors had an 8% lower adjusted risk of AD (SHR 0.92, 95% CI 0.86–0.98). However, the association disappeared in landmark analyses restricted to women surviving more than five years, arguing against a durable inverse association. Within the survivor cohort, neither tamoxifen nor aromatase-inhibitor treatment was independently associated with AD, whereas radiotherapy was associated with lower risk (13). In contrast, a Swedish register study of 26,741 five-year survivors and 249,540 cancer-free women found no overall association with all-cause dementia, AD, or vascular dementia. Women diagnosed after age 65 nevertheless had higher risks of all three outcomes, including a 35% higher risk of AD (SHR 1.35, 95% CI 1.05–1.75) (14). Together, these findings indicate that any survivorship association is modest and sensitive to age, follow-up duration, competing mortality, and survivor selection.

Studies focused on endocrine treatment are similarly heterogeneous. In a US claims-based cohort of 57,843 women with breast cancer, exposure to hormone-modulating therapy was associated with an 18% lower incidence of AD (RR 0.82, 95% CI 0.75–0.90) and a 12% lower incidence of dementia (RR 0.88, 95% CI 0.83–0.93), with the strongest inverse associations observed for tamoxifen and the steroidal aromatase inhibitor exemestane (15). A subsequent SEER–Medicare analysis of 18,808 women aged 65 years or older found a smaller 7% reduction in AD and related dementias (HR 0.93, 95% CI 0.88–0.98) over approximately 12 years. The association varied substantially by age, race, and treatment class: it was strongest among Black women aged 65–74 years and was absent among White women aged 75 years or older (3). Conversely, a UK cohort of 14,214 postmenopausal survivors found no difference in dementia risk between aromatase-inhibitor and tamoxifen users (16). Importantly, cancer-related cognitive impairment should not be equated with AD. Breast cancer chemotherapy and endocrine therapy can affect memory, attention, executive function, and processing speed, but these heterogeneous and often partly reversible changes do not establish progressive AD pathology (17). Overall, current evidence does not support a consistent association of breast cancer or estrogen-directed therapy with either lower or higher AD risk.

### 3.2 Prostate cancer: androgen-deprivation therapy

Prostate cancer extends this distinction to androgen signaling, for which the neurological consequences of profound and sustained hormone suppression may differ from any association attributable to the tumor itself. Androgen receptor signaling is a central driver of prostate cancer, whereas androgens contribute to neural, metabolic, and vascular homeostasis. Prostate cancer diagnosis and androgen-deprivation therapy (ADT) must therefore be evaluated separately. For prostate cancer itself, the apparent inverse association may reflect altered detection as much as an underlying biological association. A Swedish nationwide study compared 178,746 men with prostate cancer with 1,787,460 age-matched controls and also examined subsequent prostate cancer diagnoses among men with pre-existing dementia. Men with low- or intermediate-risk prostate cancer had little or no excess risk of AD or vascular dementia. By contrast, men with high-risk disease treated with ADT alone had higher risks of AD and vascular dementia, with hazard ratios of 1.37 and 1.51, respectively. In the reverse direction, men with pre-existing AD had substantially lower odds of subsequently diagnosed high-risk or low- to intermediate-risk prostate cancer. However, reduced prostate-specific antigen screening and diagnostic investigation after dementia may partly explain this apparent inverse association (18).

Several large observational studies associate ADT with increased dementia risk. Among 100,414 men aged ≥66 years with localized or locally advanced prostate cancer, pharmacological ADT was associated with 17% higher all-cause dementia risk and 23% higher AD risk. Exposure for ≥7 months was associated with increases of 25% and 34%, respectively (19). ADT modality may also be important. In a Taiwanese cohort of 23,651 patients, antiandrogen monotherapy was associated with increased risks of dementia and AD, whereas GnRH agonist treatment and orchiectomy showed no significant differences from no ADT (20). Other well-controlled cohorts have not reproduced these associations. Among 45,218 US veterans receiving definitive radiotherapy for nonmetastatic prostate cancer, ADT was not significantly associated with all-cause dementia, vascular dementia, or AD, including after treatment exceeding one year (21). Nevertheless, a meta-analysis of 28 studies comprising approximately 2.54 million participants found that ADT was associated with 20% higher all-cause dementia risk and 26% higher AD risk (4). The substantial variation among individual studies indicates that these pooled associations do not establish causality. Taken together, pooled observational evidence suggests a modest association between ADT exposure and all-cause dementia and AD, but substantial heterogeneity among studies precludes a causal conclusion (4). Differences in treatment modality and duration may contribute: recent meta-analyses report variation in dementia risk across ADT modalities and a nonlinear relationship with exposure duration (4, 22). A recent nationwide Korean cohort likewise found no significant association between ADT and overall dementia but a higher risk of AD, further illustrating outcome-specific heterogeneity (23). Differences in cancer stage and treatment indication may further introduce confounding by disease severity, while age and cardiovascular or metabolic comorbidities influence both ADT selection and baseline dementia risk. Finally, studies vary in follow-up duration and dementia ascertainment, ranging from administrative diagnostic codes to clinically defined outcomes, which may contribute to inconsistent estimates. Thus, current evidence supports a possible ADT-associated neurological risk rather than a protective or harmful effect attributable to prostate cancer itself. The major epidemiological studies discussed above, including differences in study population, cancer type, treatment exposure, neurological outcome, follow-up duration, and effect estimates, are summarized in **Table 1**.

**Table 1:** Selected epidemiological studies of cancer, dementia/Alzheimer disease, and endocrine treatment.

Population	Cancer type	Sample size	Exposure/treatment	Outcome/ascertainment	Key effect estimate (95% CI)	Follow-up	Major limitations	Ref
UK adults ≥60 y; both sexes	Multiple cancers	3,021,508; 412,903 with cancer	Previous/incident cancer diagnosis; MR analysis	Dementia/ADRD from EHR, hospital/death records or dementia medication	Dementia: HR 0.75 (0.74–0.76); competing-risk SHR 0.56 (0.55–0.56); genetically predicted cancer–AD: OR 0.97 (0.95–0.99)	Median 7.9 y; observation up to 30 y	Possible dementia under-reporting/misclassification; no biomarker-defined AD; missing covariates and residual confounding	(1)
International observational cohorts; mixed sex/age	Multiple cancers	16 studies; 3,615,269 cancer survivors	Cancer diagnosis; also endocrine therapy, chemotherapy, radiotherapy	All-cause dementia (ACD), AD, VaD	ACD: RR 0.92 (0.83–1.02); AD: RR 0.88 (0.78–0.99); VaD: RR 0.86 (0.75–0.99)	Varied; analyses stratified by follow-up duration	Very high between-study heterogeneity; observational evidence; English/published literature only; limited VaD evidence	(5)
UK Biobank participants ≥55 y; predominantly White	Multiple cancers	140,959	Prevalent or incident cancer	All-cause dementia, AD, VaD from linked health records	Incident cancer–all-cause dementia: HR 1.14 (1.02–1.29); prevalent cancer: HR 1.04 (0.92–1.17); AD not significantly associated; first year after cancer HR 1.83 (1.42–2.36)	Median 12.3 y	Healthy-volunteer/White-population selection; relatively young cohort; limited dementia cases; EHR diagnostic bias and incomplete time-varying health-care utilization	(6)
Rotterdam Study; adults 60–70 y, cognitively normal at baseline	Multiple cancers	3,634	Incident cancer diagnosis treated as time-varying proxy exposure	Dementia diagnosis from medical records/registries	Dementia RR ranged from 0.71 (0.49–0.95) to 1.10 (0.79–1.30), depending on exposure definition and bias-control assumptions	Up to 20 y	Primarily a causal-methodology case study; cancer is an imperfect proxy for proposed mechanisms; estimates sensitive to assumptions about death/confounding; second subcohort had 15-y follow-up extrapolated to 20 y	(7)
Community autopsy cohort; mean age at death 83.8 y; 60.1% female	Multiple cancers	785; 190 with cancer	Registry-confirmed cancer history	MCI/dementia and neuropathology at autopsy	MCI/dementia: OR 0.45 (0.32–0.65); Braak V/VI: OR 0.38 (0.26–0.55); neuritic plaques: OR 0.52 (0.36–0.75)	Longitudinal cohort; autopsy at death	Only 190 cancer cases; unable to evaluate site, stage or treatment; autopsy cohort predominantly White and well educated	(8)
Korean women undergoing breast cancer surgery; mean age 53.1 y	Breast	70,701 survivors; matched cancer-free controls	Breast cancer and subsequent treatments	AD from national claims; competing-risk models	Breast cancer vs controls: SHR 0.92 (0.86–0.98); radiotherapy: adjusted HR 0.77 (0.68–0.87); inverse association did not persist beyond 5 y	Median 7.3 y	No detailed stage/radiation-dose data; ICD-based AD ascertainment; selection and residual confounding; maximum follow-up 11 y	(13)
Swedish women ≥50 y who survived breast cancer ≥5 y	Breast	26,741 survivors; 249,540 controls	Breast cancer survivorship	All-cause dementia, AD, VaD using ICD codes	Overall association null; diagnosis after age 65: ACD SHR 1.30 (1.07–1.58), AD 1.35 (1.05–1.75), VaD 1.64 (1.11–2.43)	Up to 15 y after survivor entry	Registry codes have limited sensitivity/onset precision; less severe dementia may be missed; race/ethnicity unavailable; survivor selection	(14)
US women ≥45 y with breast cancer; mean age ≈76 y	Breast	57,843	Hormone-modulating therapy (SERMs, ER antagonists, aromatase inhibitors)	Claims-defined neurodegenerative disease, AD and dementia	AD: RR 0.82 (0.75–0.90); dementia: RR 0.88 (0.83–0.93)	Mean 5.5 y	Retrospective claims data; residual confounding; filled prescriptions do not ensure adherence; limited pathology/treatment detail	(15)
US women ≥65 y with newly diagnosed breast cancer	Breast	18,808	Hormone-modulating therapy vs no HMT	ADRD from SEER–Medicare	HMT: HR 0.93 (0.88–0.98); association varied substantially by age and race	Mean ≈12 y	Medicare population limits age generalizability; no APOE or AD biomarkers; HMT duration not modeled; residual treatment confounding	(3)
UK postmenopausal women ≥54 y with breast cancer	Breast	14,214	Aromatase inhibitor vs tamoxifen	Incident dementia from primary-care records/prescriptions	AI vs tamoxifen: HR 1.04 (0.83–1.30); no significant difference	57,102 person-years	Chemotherapy, stage, grade and ER status incompletely available; undiagnosed dementia missed; residual confounding	(16)
Swedish men with prostate cancer; median age 67 y (low/intermediate risk), 75 y (high risk)	Prostate	178,746 PC; 1,787,460 matched controls	PC diagnosis, risk category and treatment including ADT	AD and VaD from national registers	High-risk PC treated only with ADT: AD HR 1.37 (1.19–1.58); VaD HR 1.51 (1.29–1.78). Prior AD associated with lower odds of high-risk PC: OR 0.39 (0.35–0.45)	Median 7 y low/intermediate risk; 4 y high-risk	ICD-based dementia may be underdiagnosed; possible detection bias; Swedish population limits generalizability	(18)
US men ≥66 y; median age 73 y; localized/locally advanced PC	Prostate	100,414	Pharmacologic ADT; duration 0, 1–6, ≥7 mo	All-cause dementia and AD from Medicare claims	Any ADT: all-cause dementia HR 1.17 (1.07–1.27); AD HR 1.23 (1.11–1.37)	36 mo	Claims misclassification/underreporting; differential diagnostic surveillance with ADT; potential exposure-time alignment bias; relatively short follow-up	(19)
Taiwanese men; median age 73 y	Prostate	23,651	GnRH agonist, orchiectomy, antiandrogen monotherapy vs no ADT	All-cause dementia and AD from linked claims/registries	Antiandrogen: dementia HR 1.34 (1.16–1.55), AD HR 1.52 (1.13–2.04); GnRH and orchiectomy not significantly different from no ADT	Median 3.46 y	Claims-based diagnosis; residual confounding (education, lifestyle, family history); potential detection bias	(20)
US male veterans with nonmetastatic PC receiving definitive radiotherapy	Prostate	45,218	Radiotherapy with vs without ADT	Any dementia, VaD, AD	Any dementia: SHR 1.04 (0.94–1.16); VaD 1.20 (0.97–1.50); AD 1.11 (0.91–1.36); all nonsignificant	Median 6.8 y	Restricted to veterans receiving definitive radiotherapy; limited generalizability to other PC populations/treatments	(21)
International studies of men with prostate cancer	Prostate	28 studies; 2,543,483 participants reported across 27 studies	ADT vs no ADT; multiple ADT modalities/durations	Dementia, AD, VaD, PD	Dementia: HR 1.20 (1.11–1.29); AD: HR 1.26 (1.10–1.43); VaD: HR 1.30 (0.97–1.73)	Varied across included studies	Substantial methodological heterogeneity and residual confounding; differences in disease stage/treatment; possible overlapping databases	(4)

## 4 Mechanisms of inverse comorbidity

The mechanisms considered here concern tumor- and tissue-level biology and should be distinguished from population-level associations and from systemic effects of anticancer treatment. These levels need not operate in the same direction, and the pathways discussed below should therefore be interpreted as candidate biological contributors rather than direct explanations of the epidemiological inverse association.

### 4.1 Endocrine–metabolic signaling

The insulin/insulin-like growth factor (IGF)–PI3K–AKT axis provides a central metabolic intersection between cancer and AD, but its consequences depend on tissue context. In many cancers, hyperinsulinemia, IGF signaling, and oncogenic PI3K activation support glucose uptake, anabolic metabolism, proliferation, and resistance to apoptosis. Loss of PIP4K2C, for example, sensitized melanoma cells to insulin-dependent PI3K–AKT signaling and enabled them to exploit the insulin-rich liver environment for organ-specific metastasis (24). In the AD brain, by contrast, postmortem tissue shows impaired insulin-receptor–IRS-1–PI3K and IGF1-receptor–IRS-2–PI3K responses, with inhibitory IRS-1 phosphorylation correlating with Aβ pathology and cognitive decline (25). Systematic evidence further identifies brain insulin resistance and impaired glucose metabolism as recurrent features of AD, with metabolic dysfunction showing a more consistent association with tau than with amyloid biomarkers (26, 27). In rodent models, increasing circulating IGF-1 facilitated cerebral Aβ clearance by promoting the brain entry of Aβ carrier proteins, illustrating that peripheral IGF-1 actions can differ from impaired central insulin signaling (28). These shared endocrine and metabolic pathways help explain how cancer and AD can emerge from overlapping regulatory networks yet diverge into opposite biological outcomes (**Figure 1**).

**Figure 1:**
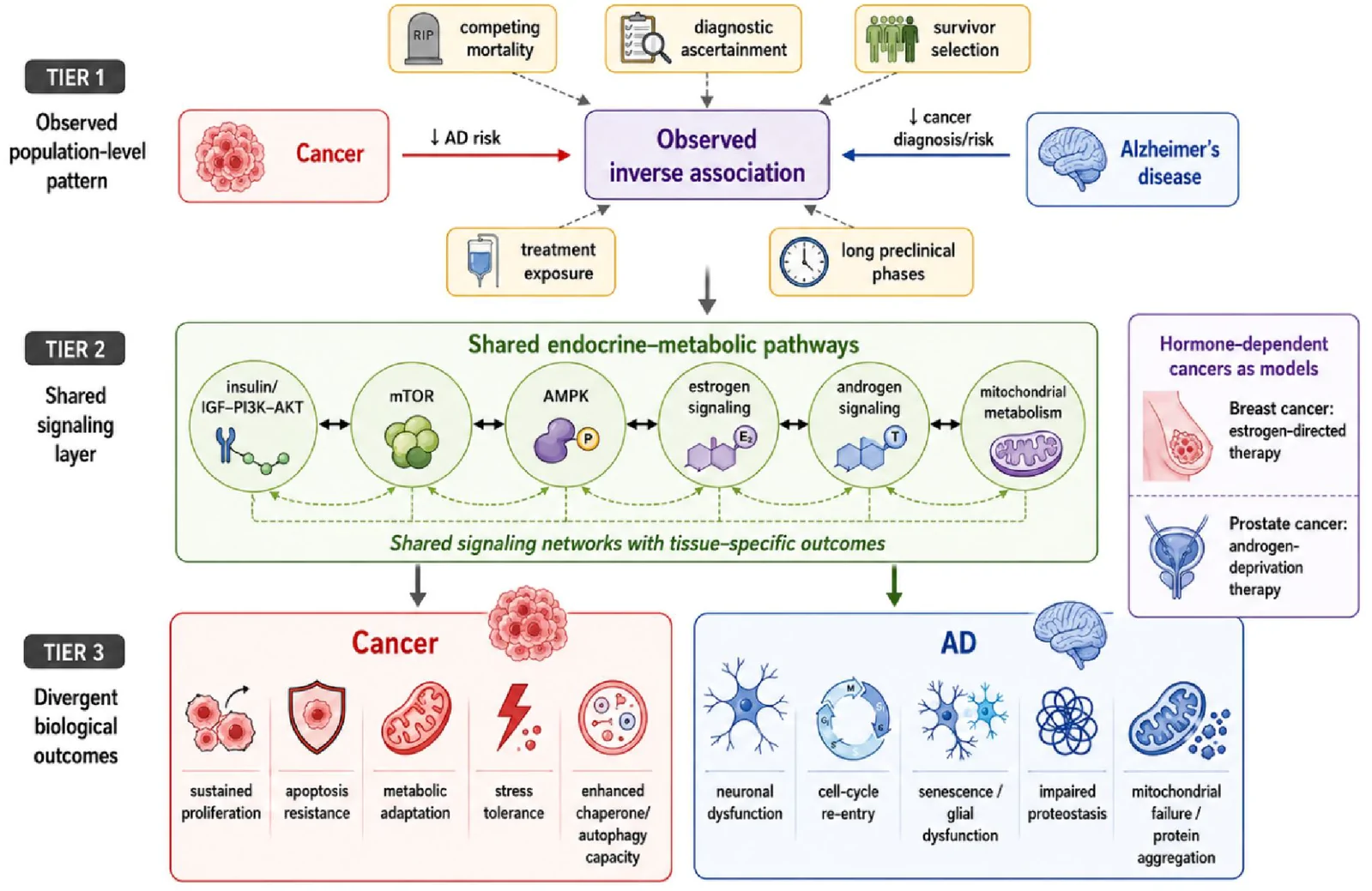
Multilevel framework for cancer–Alzheimer’s disease inverse comorbidity. (Tier 1) The observed inverse association is shaped by competing mortality, diagnostic ascertainment, survivor selection, treatment exposure, and long preclinical phases. (Tier 2) Shared endocrine–metabolic pathways—including insulin/IGF–PI3K–AKT, mTOR, AMPK, sex-steroid signaling, and mitochondrial metabolism—produce tissue-specific effects and are further modified by endocrine therapy in breast and prostate cancer. (Tier 3) These networks diverge toward proliferation, apoptosis resistance, metabolic adaptation, and enhanced stress-buffering capacity in cancer, but toward neuronal dysfunction, aberrant cell-cycle re-entry, senescence, impaired proteostasis, and mitochondrial failure in AD. The epidemiological association in Tier 1 is supported by human observational evidence, whereas many pathway-level connections in Tiers 2–3 derive from human tissue studies and experimental models; their contribution to population-level inverse comorbidity remains inferential.

The mechanistic target of rapamycin (mTOR) promotes protein synthesis and biosynthetic growth in tumors, whereas dysregulated mTOR activity in AD may aggravate metabolic and proteostatic stress. Rapamycin enhanced synaptic and mitochondrial metabolism in AD-model mice, but produced distinct responses in APOE3 and APOE4 backgrounds, indicating that the consequences of mTOR inhibition depend on genetic and metabolic context (29). AMP-activated protein kinase (AMPK) can restrain anabolic mTOR signaling, yet established tumors may exploit AMPK to survive nutrient limitation. Breast cancer cells adapted to glutamine deprivation through AMPK-dependent upregulation of PSAT1 and serine-pathway-derived α-ketoglutarate (30), while AMPK-driven phosphorylation of INF2 promoted mitochondrial fission and growth in endometrial cancer (31). Conversely, activation of neuronal and microglial AMPK by GLP-1 receptor agonists reduced amyloidogenic APP processing, enhanced Aβ phagocytosis, and improved memory in AD-model mice (32).

Mitochondrial metabolism provides a parallel distinction between tumor adaptation and neuronal energetic failure. In aging and amnestic mild cognitive impairment, synaptic and intramitochondrial Aβ42 accumulation correlated with impaired synaptic mitochondrial bioenergetics (33). AD brain tissue also exhibited increased methylation of mitochondrial ND5 mRNA, reduced complex I expression, and impaired respiration (34). Restoring NAD^+^ with nicotinamide mononucleotide enhanced the ATF4-dependent mitochondrial stress response and reduced synaptic and neuronal injury across AD models, further implicating loss of metabolic reserve in neurodegeneration (35). Sex-steroid signaling intersects with these metabolic pathways rather than acting independently. Particularly after menopause, obesity and insulin resistance increase estrogen availability and insulin–PI3K–AKT–mTOR signaling and are associated with poorer outcomes and possible endocrine-therapy resistance in ER-positive breast cancer (36). In neural systems, estrogen supports glucose utilization, mitochondrial respiration, and ATP production, although these effects depend on age, timing, and the metabolic health of the target cells (37). In prostate cancer, reciprocal AR–IGF1 signaling supports both androgen-dependent and androgen-independent growth through epithelial and stromal mechanisms (38). Taken together, these data establish that cancer and AD engage overlapping endocrine–metabolic networks, but they do not demonstrate that these pathways generate the epidemiological inverse association. Their contribution remains context-dependent and inferential, particularly because endocrine therapy can perturb the same pathways independently of tumor biology.

### 4.2 Cell fate and chaperone-regulated proteostasis

Cancer cells bypass checkpoint arrest and apoptosis to sustain proliferation, whereas post-mitotic neurons cannot complete productive cell division. In aging and AD, persistent DNA-damage responses can reactivate cell-cycle machinery and drive neurons toward senescence or degeneration (39). Aβ oligomers can induce tau-dependent neuronal cell-cycle re-entry (40). Under oxidative stress, tau promotes S-phase entry as a transient pro-survival response (41), whereas single-cell analyses link neuronal cell-cycle re-entry more broadly to a senescent state (42). PIN1 illustrates how the same conformational regulator can promote tumor growth while preserving neuronal protein homeostasis. In breast cancer, PIN1 prevents the ubiquitin-dependent proteasomal and lysosomal degradation of METTL3, thereby increasing m^6^A-dependent translation of TAZ and EGFR and promoting tumorigenesis (43). Conversely, reduced PIN1 expression was detected in neocortical and limbic regions of individuals with mild cognitive impairment or AD (44), particularly in women, and was associated with greater neurofibrillary tangle burden and poorer cognition.

Cellular senescence is similarly bidirectional. Senescence initially restrains abnormal proliferation, but persistent senescent cells acquire a senescence-associated secretory phenotype that can promote chronic inflammation, tumor progression, and age-related tissue dysfunction (45). In postmortem AD brains, imaging and single-nucleus transcriptomics of more than 200,000 nuclei revealed increased senescence markers predominantly in glia; senescent microglia were associated with greater Aβ burden and reduced expression of phagocytic pathways required for amyloid clearance (46). A pilot trial of dasatinib plus quercetin demonstrated cerebrospinal-fluid penetration of dasatinib and short-term feasibility, although the small sample did not establish cognitive efficacy (47). The HSF1–heat-shock-protein system links cellular stress adaptation with proteostasis capacity. In breast cancer cells, BAG1 overexpression increased HSF1 phosphorylation and elevated HSP70 and HSP27, supporting survival and stabilization of tumor-promoting signaling; HSP90 abundance itself was not altered (48). By chaperoning steroid hormone receptors, including ER and AR, and stabilizing diverse oncogenic clients, HSP90 contributes to the non-oncogene dependence of hormone-driven tumors (49). HSF1 also acts beyond cancer cells: its activation in tumor-infiltrating natural killer cells directly suppresses cytotoxicity and cytokine production, thereby weakening antitumor immunity (50). In the aging brain, by contrast, the stress-inducible HSF1–HSP response becomes attenuated, reducing chaperone reserve and increasing vulnerability to Aβ, tau, and other aggregation-prone proteins (51–53).

Autophagy–lysosome function determines whether proteotoxic stress is successfully buffered or progresses toward aggregate accumulation. Although autophagy can limit early tumor development, established cancers exploit it for nutrient recycling, mitochondrial quality control, metabolic adaptation, and survival under hypoxia or therapeutic stress (54, 55). In tauopathy, disruption of TFEB–vacuolar ATPase signaling impaired lysosomal acidification, increased tau pathology, and weakened the microglial response (56). APOE4 can further suppress transcription of lysosomal-autophagy genes, whereas blocking this interaction restores aggregate-clearance pathways in cellular and animal models (57). Autophagy is also non-uniform in AD: autophagic induction may be preserved or increased while downstream lysosomal degradation becomes inefficient. In AD mouse models, impaired vATPase-dependent autolysosomal acidification precedes extracellular amyloid deposition and is accompanied by accumulation of Aβ and APP-βCTF in poorly acidified autolysosomes (58). Thus, rather than a simple opposition between enhanced autophagy in cancer and impaired autophagy in AD, the consequences of autophagy depend on disease stage and the integrity of downstream lysosomal degradation. Autophagy can suppress early tumorigenesis yet support established tumors, while in AD increased autophagic activity may coexist with ineffective lysosomal clearance (**Figure 1**). More broadly, these studies support a contrast between enhanced stress tolerance and proteostatic adaptation in established tumors and progressive cell-fate and proteostasis failure in AD. However, most evidence derives from disease-specific human tissue or experimental models rather than direct cancer–AD systems. These pathways therefore provide biological plausibility for divergent cellular states, but they do not by themselves establish a mechanism for inverse comorbidity.

## 5 Amyloidogenic hormones and self-assembly at the cancer–brain interface

Protein self-assembly provides a potential mechanistic bridge between endocrinology, cancer, and AD, although the evidence ranges from direct experimental observations to still-unproven connections with population-level inverse comorbidity. Rather than being exclusively pathological, amyloid-like conformations can support the dense and stable storage of peptide and protein hormones within secretory granules (**Figure 2A**). Cross-β-rich assemblies have been detected for prolactin, adrenocorticotropic hormone, growth hormone, β-endorphin, oxytocin, and vasopressin, and these deposits can release biologically active monomers following secretion into the extracellular environment (59–61). This physiological use of controlled aggregation places endocrine tissues near a boundary between reversible hormone storage and pathological deposition. IAPP was originally identified in amyloid isolated from a pancreatic islet-cell tumor and is co-stored and co-secreted with insulin, whereas full-length calcitonin constitutes the amyloid deposited in medullary thyroid carcinoma (62, 63). Pathogenic provasopressin mutants likewise form amyloid-like assemblies in familial neurohypophyseal diabetes insipidus, linking abnormal hormone self-assembly to defective secretory-granule sorting and endocrine dysfunction (64) (**Figure 2A**). The presence of calcitonin-derived amyloid in human medullary thyroid tumors has also enabled selective imaging with an amyloid-binding PET tracer, demonstrating that hormone-derived amyloid is a clinically detectable component of an endocrine tumor microenvironment (65).

**Figure 2:**
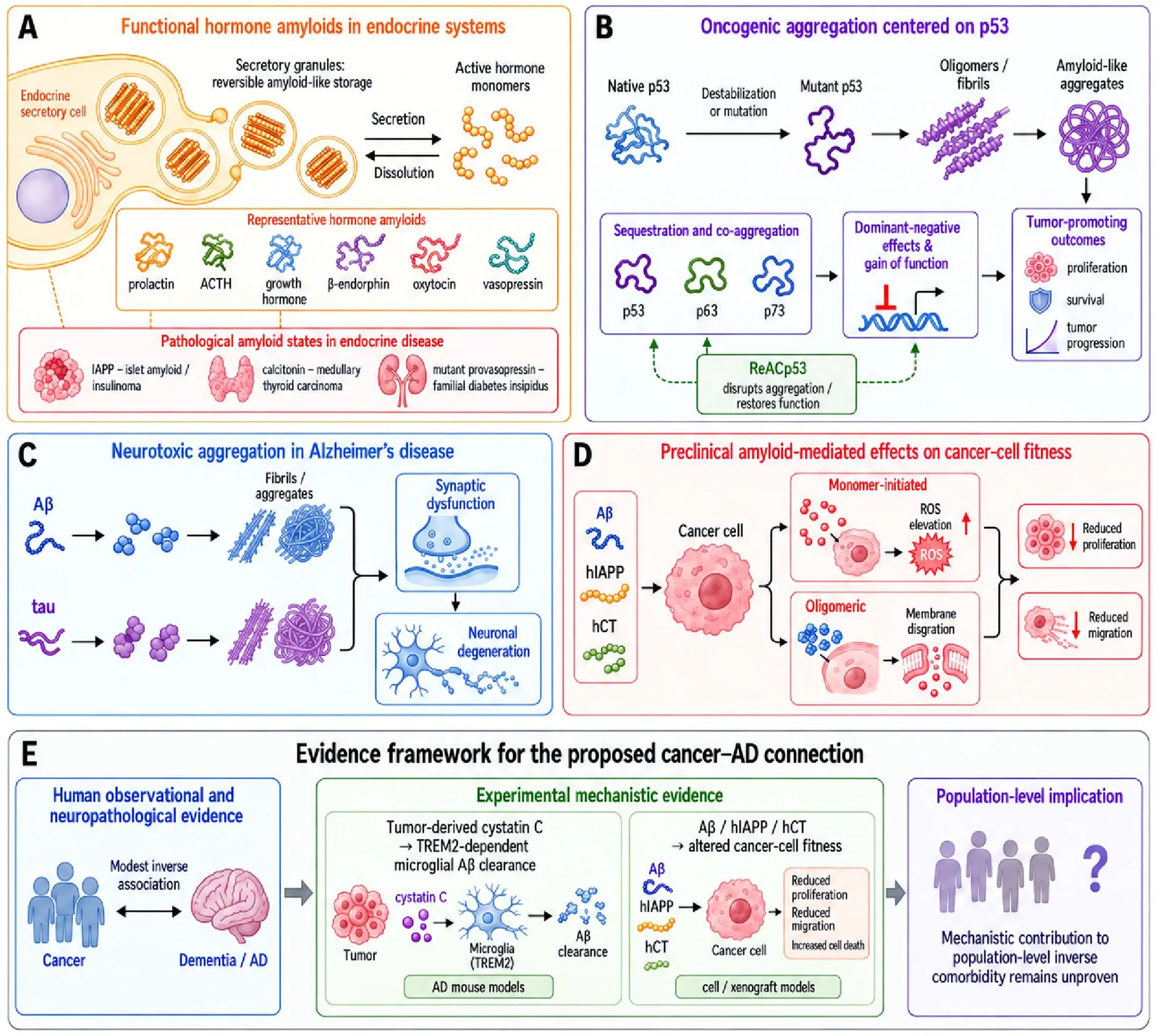
Amyloidogenic hormones and context-dependent protein self-assembly in cancer and Alzheimer’s disease. Protein self-assembly has distinct physiological and pathological consequences in endocrine tissues, cancer, and Alzheimer’s disease (AD). **(A)** Functional amyloid-like assemblies can reversibly store peptide and protein hormones in secretory granules, placing endocrine tissues near the boundary between physiological and pathological aggregation; examples include pituitary hormones, islet amyloid polypeptide (IAPP), calcitonin, and mutant provasopressin. **(B)** In cancer, aggregation of destabilized or mutant p53 can sequester wild-type p53 and its paralogs p63 and p73, producing dominant-negative and gain-of-function effects that promote tumor progression. **(C)** In AD, aggregation of Aβ and tau is associated with synaptic dysfunction and neuronal degeneration. **(D)** Preclinical studies show that Aβ, hIAPP, and human calcitonin can alter cancer-cell proliferation, migration, and viability under defined experimental conditions; these findings should not be generalized to a clinically established anticancer effect. **(E)** Evidence framework for the proposed cancer–AD connection. The inverse cancer–AD association is supported primarily by human observational and neuropathological evidence, whereas tumor-derived cystatin C–TREM2-mediated Aβ clearance and amyloid-induced alterations in cancer-cell fitness are supported by experimental models. Whether these mechanisms contribute to population-level inverse comorbidity in humans remains unproven.

Importantly, amyloidogenic peptides and their assembly states are not biologically equivalent. Molecular-dynamics simulations of Aβ- and IAPP-derived fragments showed distinct homotypic aggregate architectures and heterotypic Aβ–IAPP assemblies with altered β-barrel propensity, while Aβ42–hIAPP co-oligomers produced a more advanced AD-like phenotype with markedly increased neuronal death in human cerebral organoids (66, 67). These findings illustrate that the biological consequences of self-assembly depend on peptide identity, assembly state, and heterotypic interactions; functional hormone-storage assemblies therefore should not be equated with disease-associated toxic species. In cancer, aggregation of tumor suppressors may instead promote malignant survival. Destabilized wild-type or mutant p53 can form amyloid-like oligomers and fibrils in breast-cancer models and glioblastoma, sequestering wild-type p53 and its paralogs p63 and p73 to produce dominant-negative and gain-of-function phenotypes (68–71) (**Figure 2B**). Recent experiments further showed that oncogenic p53 converts p63 and p73 condensates into β-sheet-rich aggregates (72). Nevertheless, this behavior should be described cautiously: some studies support seeded, prion-like conformational conversion, whereas others indicate that mutant and wild-type proteins primarily associate through direct coaggregation rather than classical catalytic seeding (73). The functional importance of the aggregated state is supported by ReACp53, a designed peptide that inhibited p53 aggregation, restored p53-dependent transcription and apoptosis in ovarian carcinoma cells and patient-derived organoids, and suppressed tumor growth *in vivo (*74) (**Figure 2B**). Thus, p53 aggregation is included here as an independent cancer-side example of context-dependent protein self-assembly rather than as a mechanistic bridge between cancer and AD. We therefore do not interpret p53 aggregation as evidence for a cancer-to-AD pathway or an amyloid-mediated protective effect against cancer. Its relevance is conceptual: protein self-assembly can produce distinct pathological outcomes depending on protein identity and cellular context, as illustrated by loss or gain of p53 function in cancer versus neurotoxicity of Aβ and tau in AD. In AD, aggregation of Aβ and tau is associated with synaptic dysfunction and neuronal degeneration (**Figure 2C**). Recent evidence provides a more direct cancer-to-AD mechanistic connection. In AD mouse models, peripheral lung, prostate, and colon tumors attenuated amyloid pathology and improved cognition through secretion of cystatin C, which interacted with Aβ and activated TREM2-dependent microglial clearance. Genetic disruption of TREM2 or mutation of cystatin C abolished these effects, establishing a causal tumor-to-brain mechanism in experimental models. Notably, this effect was observed across lung, prostate, and colon tumor models, suggesting that the mechanism is not restricted to hormone-dependent cancers, although its relevance across human cancer types remains unknown. However, whether this pathway contributes to the epidemiological cancer–AD association in humans remains unknown (75).

Conversely, preclinical studies indicate that neurodegenerative and endocrine amyloids can alter cancer-cell fitness. Aβ inhibited angiogenesis and reduced the vascularization and growth of glioblastoma and lung-cancer xenografts, while an Aβ-derived antiangiogenic fragment suppressed breast-tumor growth (76, 77). Oligomeric Aβ also preferentially impaired breast cancer stem cells by disrupting lysosomal iron homeostasis and increasing iron-dependent oxidative stress, whereas tumor-targeted KLVFF motifs assembled into extracellular nanofiber networks that restricted breast-cancer migration, vascular passage, and spontaneous metastasis (78, 79). Extending these observations beyond Aβ, hIAPP, and human calcitonin inhibited PANC-1 proliferation and migration in sequence-, concentration-, and aggregation-state-dependent manners (80) (**Figure 2D**). For hIAPP, aggregation is strongly sequence- and processing-dependent: β-sheet-prone regions include residues 12–17, 23–27, and 32–37, with the FGAIL motif contributing prominently to amyloid formation; moreover, unprocessed prepro-IAPP can also aggregate and induce neuronal degeneration *in vivo (*81, 82). Comparable short amyloidogenic/self-recognition motifs have been identified in human calcitonin (DFNKF) and Aβ (KLVFF) (83, 84). These findings emphasize that biological effects observed for a particular peptide preparation should not be generalized across monomeric, oligomeric, fibrillar, intracellular, or extracellular states. Monomer-initiated conditions predominantly elevated intracellular reactive oxygen species, whereas oligomeric preparations produced greater membrane leakage and membrane-disruption-associated cell death.

The physiological relevance and cellular selectivity of these anticancer effects remain uncertain. In the PANC-1 study, amyloid peptides were tested over concentrations of 0.1–160 μM, from which IC25, IC50, and IC75 values were determined; whether comparable concentrations and aggregation states occur within human tumor microenvironments has not been established (80). Circulating Aβ concentrations are orders of magnitude below the concentrations typically used in these cell-based assays, and local concentrations within tumor microenvironments have not been established (85, 86). Moreover, experimentally prepared monomeric and oligomeric species may not reproduce the heterogeneous and transient assemblies present *in vivo*. Evidence for cancer-cell selectivity is also limited: oligomeric Aβ preferentially inhibited breast cancer stem cells relative to bulk cancer cells (79), but this does not establish selective toxicity relative to matched nonmalignant cells, and ROS generation and membrane disruption can represent general proteotoxic mechanisms. A potential basis for differential susceptibility exists because many malignant cells expose phosphatidylserine and other anionic components on the outer membrane, features exploited by some membrane-active anticancer peptides (87, 88). Whether these properties account for the effects of Aβ, hIAPP, or calcitonin remains untested. Human circulating-Aβ data also do not support a simple systemic protective model: plasma Aβ40 and Aβ42 were elevated in a case-control study of patients with several cancers, and higher plasma Aβ40 and Aβ42 were associated with higher subsequent cancer incidence in a population-based cohort (85, 86). These observations do not address local peptide concentration or aggregation state within tumors, but they further argue against interpreting circulating Aβ as a straightforward anticancer factor. These limitations generate specific testable predictions. If amyloid self-assembly contributes biologically to cancer suppression, inhibitory effects should persist at concentrations measured in relevant tumor microenvironments, vary reproducibly with aggregation state, be attenuated when aggregation is prevented, scale with outer-leaflet phosphatidylserine exposure if membrane-mediated, and show greater effects in malignant cells than in matched nonmalignant cells. Failure to meet these criteria would favor nonspecific proteotoxicity rather than a cancer-selective mechanism.

Together, these preclinical studies show that protein self-assembly can influence both neurodegenerative and cancer phenotypes in experimental systems, but they do not establish that amyloid-mediated suppression of cancer-cell fitness explains inverse comorbidity in humans. Indeed, melanoma-secreted Aβ can promote brain metastasis by suppressing local neuroinflammatory responses, further demonstrating that amyloid effects are strongly context dependent (85, 89). Thus, the contribution of amyloid self-assembly to the population-level cancer–AD association should presently be regarded as a testable mechanistic hypothesis rather than an established causal mechanism. The major metabolic, cell-fate, proteostatic, and protein-self-assembly mechanisms discussed above are summarized in **Table 2**.

**Table 2:** Major mechanistic pathways at the cancer–Alzheimer’s disease interface.

Pathway/protein	Cancer context	AD context	Key mechanistic connection	Experimental model
Insulin/IGF–PI3K–AKT	Supports glucose uptake, anabolic metabolism, proliferation, and apoptosis resistance	Brain insulin resistance and impaired insulin/IGF signaling	Shared metabolic network produces divergent tissue-specific outcomes	Cancer cell models; human AD brain tissue; rodent models
mTOR/AMPK	Supports biosynthesis and metabolic adaptation under stress	Modulates neuronal metabolism, APP processing, Aβ clearance, and proteostasis	Nutrient-sensing pathways influence adaptation versus dysfunction	Cancer cell models; AD mouse models
Mitochondrial metabolism	Enables metabolic flexibility and survival under stress	Mitochondrial energetic failure and oxidative stress	Different capacities to maintain metabolic reserve under chronic stress	Human AD tissue; cellular and animal models
Cell cycle/PIN1/senescence	Promotes proliferation and tumor progression; senescence may support tumor-promoting inflammation	Aberrant neuronal cell-cycle re-entry and glial senescence contribute to neurodegeneration	Opposing regulation of cell fate and stress responses	Cancer cell models; human AD tissue; single cell/transcriptomic studies
Chaperones/autophagy–lysosome	Supports proteotoxic stress tolerance and survival in established tumors	Reduced chaperone reserve and inefficient lysosomal degradation promote aggregate accumulation	Proteostasis capacity determines whether misfolded proteins are cleared, tolerated, or become pathogenic	Cancer cell models; human AD tissue; tauopathy and AD animal models
p53 aggregation	Aggregated p53 can produce dominant-negative and gain-of-function phenotypes that promote malignancy	No established direct AD pathway	Illustrates context-dependent pathological consequences of protein self-assembly	Cancer cells; patient-derived organoids; tumor models
Cystatin C–TREM2	Peripheral tumors secrete cystatin C	Promotes TREM2-dependent microglial Aβ clearance	Direct tumor-to-brain signaling pathway affecting amyloid pathology	Lung, prostate, and colon tumor models in AD mice
Aβ/hIAPP/calcitonin	Can alter angiogenesis, proliferation, migration, and cancer-cell fitness	Amyloid assembly contributes to neurodegenerative pathology	Effects depend on peptide identity, concentration, and aggregation state	Cancer cell assays; xenografts; peptide-assembly models

## 6 Conclusions and future outlook

The inverse association between cancer and dementia, including AD, is unlikely to arise from a single causal pathway. Rather, the observed pattern likely reflects the combined effects of competing mortality, diagnostic ascertainment, systemic endocrine and metabolic states, anticancer treatment, and oppositely regulated cellular programs. Hormone-dependent cancers illustrate this complexity: tumor-associated proliferative and metabolic states may differ fundamentally from the systemic consequences of endocrine therapy. Accordingly, breast cancer hormone-modulating therapy and prostate cancer androgen-deprivation therapy can show distinct neurological associations. Genetic and transcriptomic studies further identify opposing associations across AD and several cancers, implicating cell-cycle control, apoptosis, metabolism, and proteostasis. This framework is centered on Alzheimer-type amyloid–tau pathology and should not be assumed to generalize to other neurodegenerative proteinopathies. Cancer associations differ across disorders—for example, melanoma shows a positive association with Parkinson’s disease—and neuropathological associations with TDP-43 and α-synuclein appear less consistent than those observed for AD-type pathology. Preclinical evidence that amyloid assemblies can suppress aspects of cancer-cell fitness under defined experimental conditions provides biological plausibility for one connection, while the recently identified tumor-derived cystatin C–TREM2 pathway establishes a cancer-to-brain mechanism in mice; whether either contributes materially to inverse comorbidity in humans remains unknown.

Future studies should therefore separate cancer subtype and stage from endocrine treatment class, dose, and duration while applying time-varying exposure and competing-risk analyses. Incorporating plasma Aβ_42_/Aβ_40_ ratio, phosphorylated tau, neuroimaging, endocrine markers, and metabolic profiles could determine whether cancer or its treatment modifies preclinical AD biology rather than only dementia diagnosis. Mechanistic studies should test amyloid–cancer interactions at physiologically relevant concentrations, aggregation states, and exposure durations in patient-derived organoids, immune-competent cocultures, and animal models. Therapeutic interpretation must remain cautious: pathways revealed by inverse comorbidity may identify useful targets, but neither amyloid toxicity nor anticancer treatment should be considered intrinsically beneficial. Integrating treatment-aware epidemiology with physiologically relevant models will be essential to distinguish genuine biological antagonism from observational bias.
